# Association of Low Serum Albumin Level with Higher Hepatocellular Carcinoma Recurrence in Patients with Hepatitis B Virus Pre-S2 Mutant after Curative Surgical Resection

**DOI:** 10.3390/jcm10184187

**Published:** 2021-09-16

**Authors:** Long-Bin Jeng, Tsai-Chung Li, Shih-Chao Hsu, Wen-Ling Chan, Chiao-Fang Teng

**Affiliations:** 1 Organ Transplantation Center, China Medical University Hospital, Taichung City 404, Taiwan; longbin.cmuh@gmail.com (L.-B.J.); quietlotus@gmail.com (S.-C.H.); 2 Department of Surgery, China Medical University Hospital, Taichung City 404, Taiwan; 3 School of Medicine, China Medical University, Taichung City 404, Taiwan; 4 Department of Public Health, College of Public Health, China Medical University, Taichung City 404, Taiwan; tcli@mail.cmu.edu.tw; 5 Department of Healthcare Administration, College of Medical and Health Science, Asia University, Taichung City 404, Taiwan; 6 Department of Bioinformatics and Medical Engineering, Asia University, Taichung City 404, Taiwan; wlchan@asia.edu.tw; 7 Epigenome Research Center, China Medical University Hospital, Taichung City 404, Taiwan; 8 Graduate Institute of Biomedical Sciences, China Medical University, Taichung City 404, Taiwan; 9 Research Center for Cancer Biology, China Medical University, Taichung City 404, Taiwan

**Keywords:** hepatocellular carcinoma, hepatitis B virus, pre-S2 mutant, serum albumin level, recurrence

## Abstract

Hepatocellular carcinoma (HCC) is, globally, one of the most prevalent and deadly human cancers; despite curative surgical resection, its high recurrence rate after surgery remains a large threat, resulting in poor patient survival. The hepatitis B virus (HBV) pre-S2 mutant that harbors deletions spanning the pre-S2 gene segment has emerged as an important oncoprotein for HCC development and a valuable prognostic biomarker for HCC recurrence; however, its relationship with clinicopathological factors is largely unexplored. In this study, the correlation of the deletion spanning the pre-S2 gene segment with clinicopathological factors and the association of such correlation with HCC recurrence after curative surgical resection were examined in HBV-related HCC patients. Inverse correlation between serum albumin level and the deletion spanning the pre-S2 gene segment was identified. HCC patients with the presence of the deletion spanning the pre-S2 gene segment and a low serum albumin level were associated with higher HCC recurrence than patients with either factor alone or neither factor were. Moreover, a combination of the serum albumin level and the deletion spanning the pre-S2 gene segment exhibited better performance than that of either factor alone in predicting HCC recurrence. Collectively, this study shows an association of low serum albumin level with pre-S2 mutant-positive HCC patients, and validates the prognostic value of this association in identifying patients with higher HCC recurrence after curative surgical resection.

## 1. Introduction

Hepatocellular carcinoma (HCC) is the dominant type of liver cancer and, globally, is the sixth most frequent and third most lethal human cancer, causing up to 800,000 deaths every year [1,2]. Although liver transplantation and surgical resection are available as potentially curative treatments for HCC, the former is limited by a scarcity of donor livers, and the latter is challenged by high HCC recurrence after surgery, leading to poor patient survival [3,4]. Moreover, the high drug resistance and genetic heterogeneity of HCC limit the survival benefits of chemotherapy and molecular targeted therapy in HCC patients, respectively [5,6]. Therefore, it is still a key goal to discover valuable biomarkers and therapeutics of HCC development and recurrence for early detection and better management to improve patient outcomes.

Chronic hepatitis B virus (HBV) infection is intimately associated with HCC development and globally accounts for over 50% of total HCC cases [7,8]. The pre-S2 mutant is a naturally occurring mutant of the HBV large-surface protein, which is encoded by an HBV surface gene that harbors deletion mutations in the pre-S2 gene segment [9,10]. The pre-S2 mutant plays an important role in HBV-related HCC development through activating multiple oncogenic signaling pathways to promote hepatocyte survival, proliferation, and genomic instability in vitro and in vivo [11,12]. Pre-S2 mutant-activated signaling pathways are regarded as potential therapeutic targets for HBV-related HCC [13,14,15]. Furthermore, the presence of the pre-S2 mutant in liver tissue or blood is a good independent biomarker for a higher risk of HCC development and recurrence after curative surgical resection [16,17,18,19,20,21,22,23,24]. However, the underlying pathological mechanisms of HCC recurrence in patients with pre-S2 mutant remain to be clarified, and hold promise to develop potential biomarkers and therapeutic targets for this high-risk population.

In this study, 75 HBV-related HCC patients who had received curative surgical resection were enrolled and classified into pre-S2 mutant-positive and -negative groups according to the presence and absence of the deletion spanning the pre-S2 gene segment in blood, respectively. The clinicopathological factor, which was correlated with the deletion spanning the pre-S2 gene segment, was identified, and its association with HCC recurrence in pre-S2 mutant-positive HCC patients after surgery was evaluated.

## 2. Materials and Methods

### 2.1. Patient Specimen and Clinicopathological Data

Plasma samples were retrospectively collected from 75 HBV-related HCC patients on the day of curative surgical resection that they received at China Medical University Hospital (Taichung, Taiwan), from March 2004 to September 2016, under the approval of the China Medical University and Hospital Research Ethics Committee (protocol code CMUH107-REC1-080; date of approval: 19 July 2018). Several clinical and pathological characteristics were obtained from the patients, including age, gender, smoking history, alcohol consumption, hepatitis B viral factors, liver-specific factors, liver disease scores, tumor properties, and tumor staging systems, due to their close association with HBV-related liver disease progression. All research was performed in accordance with the guidelines of the Declaration of Helsinki, and informed consent was obtained from all participants.

### 2.2. Detection of HBV Pre-S2 Mutant in Blood

Pre-S2 mutant in the blood of HBV-related HCC patients was detected with a next-generation sequencing (NGS)-based method as described [25]. Briefly, the HBV pre-S gene (composed of the pre-S1 and pre-S2 gene segments) was amplified from plasma DNA by polymerase chain reaction, followed by NGS analysis (Illumina, San Diego, CA, USA), to determine the percentage of wild-type and three mutant forms of the pre-S gene (including pre-S1, pre-S2, and pre-S1 + pre-S2 deletions). The presence of the deletion spanning the pre-S2 gene segment was defined as the percentage of either pre-S2 or pre-S1 + pre-S2 deletion above a cut-off of 4.643%. This cut-off percentage has been validated to provide highly accurate detection of pre-S gene deletions in the NGS-based pre-S genotyping analysis for the prediction of HCC recurrence [22,25]. On the basis of the presence and absence of the deletion spanning the pre-S2 gene segment in blood, patients were classified into pre-S2 mutant-positive and -negative groups, respectively. The pre-S genotyping results of the patients enrolled in this study are summarized in Table S1.

### 2.3. Statistical Analysis

Correlation between the deletion spanning the pre-S2 gene segment or serum albumin level and clinicopathological factors was assessed with the chi-squared test. Univariate and multivariate recurrence-free survival (RFS) analyses were performed with the Cox proportional-hazards regression model. RFS curves were analyzed with the Kaplan–Meier method and compared with the log-rank test. The receiver operating characteristic (ROC) curves of prognostic factors were used to distinguish patients with HCC recurrence from those without, and the area under the ROC curve (AUC) was calculated and compared with the Hanley–McNeil test.

## 3. Results

### 3.1. Clinicopathological Profile of Patients and Classification by Pre-S2 Mutant

As summarized in Table 1, among the 75 HBV-related HCC patients enrolled in this study, 68 (91%) were men and 7 (9%) were women; the median age was 53 years (range, 26 to 78); 60 (80%) had genotype B and 15 (20%) had genotype C HBV infection; the medium HBV DNA level was 2.1 × 10^4^ copies/mL (range, 21.5 to 1.5 × 10^8^); 65 had available hepatitis B surface antigen (HBsAg) data, and all were positive; 71 had available hepatitis B e antigen (HBeAg) data, and 62 (83%) were negative; median tumor size was 4.5 cm (range, 1.1 to 19.5). All patients received curative surgical resection, among which 52 (69%) suffered HCC recurrence after surgery; the median RFS was 11.2 months (range, 1.5 to 72.3). Moreover, among all patients, 31 (41%) were positive for deletion spanning the pre-S2 gene segment in blood and were defined as the pre-S2 mutant-positive patients; conversely, the other 44 (59%) were negative for deletion spanning the pre-S2 gene segment in blood and were defined as the pre-S2 mutant-negative patients.

### 3.2. Association of Low Serum Albumin Level with Pre-S2 Mutant-Positive HCC Patients

To identify the clinicopathological factor that was associated with pre-S2 mutant-positive HCC patients, the correlation between the deletion spanning the pre-S2 gene segment and several clinicopathological factors was examined. As shown in Table 2, among all the analyzed clinicopathological factors, the serum albumin level was the only one that displayed significant correlation with the deletion spanning the pre-S2 gene segment; pre-S2 mutant-positive HCC patients were significantly associated with a higher proportion of low serum albumin levels (≤3.8 g/dL; 23 of 31 (74%) patients) than that of pre-S2 mutant-negative HCC patients (23 of 44 (52%) patients) (*p* value = 0.0311).

### 3.3. Association of Low Serum Albumin Level with Higher HCC Recurrence in Pre-S2 Mutant-Positive HCC Patients after Curative Surgical Resection

**Figure 1:**
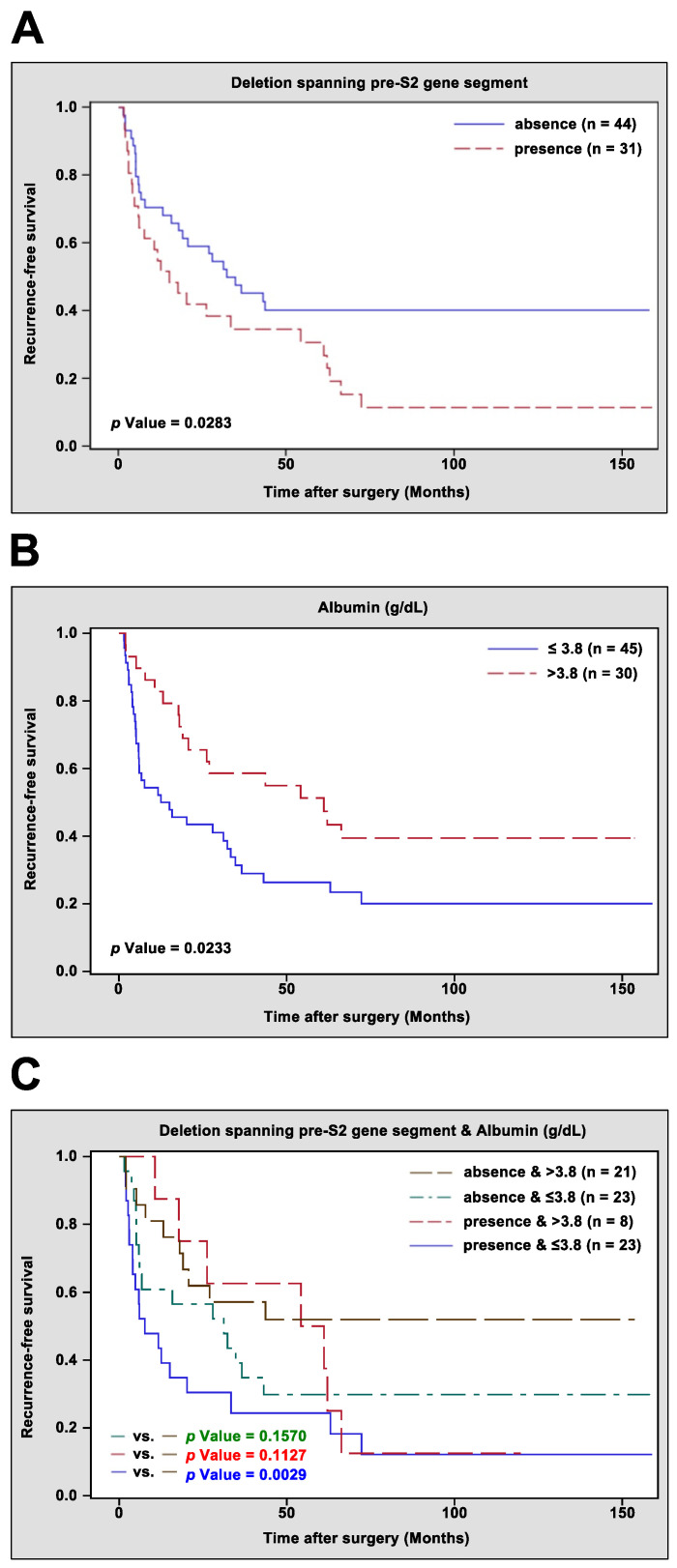
Kaplan–Meier curves for comparing the RFS difference between different groups of HBV-related HCC patients after curative surgical resection. (**A**) RFS difference between patients with the presence and absence of deletion spanning the pre-S2 gene segment. (**B**) RFS difference between patients with high (>3.8 g/dL) and low (≤3.8 g/dL) serum albumin level. (**C**) RFS difference between patients with the presence or absence of deletion spanning the pre-S2 gene segment in combination with high or low serum albumin level. Statistical significance of RFS difference shown in the lower left corner.

To assess the correlation between the deletion spanning the pre-S2 gene segment, serum albumin level, and other clinicopathological factors and HCC recurrence, univariate and multivariate analyses were performed, and RFS curves were established. As shown in Table 3 and Figure 1A, consistent with previous results [22], the presence of the deletion spanning the pre-S2 gene segment was significantly associated with poorer RFS than the absence of the deletion spanning the pre-S2 gene segment was (median RFS, 8.5 vs. 32.9 months, *p* value = 0.0283). Among analyzed clinicopathological factors, a high Child–Pugh cirrhosis score (B/C) and high AJCC TNM stage (IIIA/IIIB/IIIC/IVA/IVB) had a significantly negative impact on RFS (Table 3); conversely, high serum albumin level showed a significantly positive impact on RFS (Table 3) and was associated with better RFS than low serum albumin level was (median RFS, 19.0 vs. 6.0 months, *p* value = 0.0233) (Figure 1B). Furthermore, multivariate analysis of these four factors, which showed significance with RFS in univariate analysis, revealed that the deletion spanning the pre-S2 gene segment and AJCC TNM stage were independent prognostic factors for HCC recurrence; however, serum albumin level and Child–Pugh cirrhosis score were not (Table 3). When the serum albumin level was excluded from multivariate analysis, the significance of the AJCC TNM stage with RFS was similarly noticeable, but the significance of the deletion spanning the pre-S2 gene segment with RFS became more evident, and the association of Child–Pugh cirrhosis score with RFS changed from insignificant to significant, suggesting a dependent relationship between the serum albumin level, Child–Pugh cirrhosis score, and deletion spanning the pre-S2 gene segment. Indeed, further analysis showed significant correlation between serum albumin level and Child–Pugh cirrhosis score; HCC patients with low serum albumin level were significantly associated with a higher Child–Pugh cirrhosis score (B/C; 16 of 46 (35%) patients) than patients with a high serum albumin level (2 of 29 (3%) patients) (*p* value = 0.0042) (Table 4).

**Figure 2:**
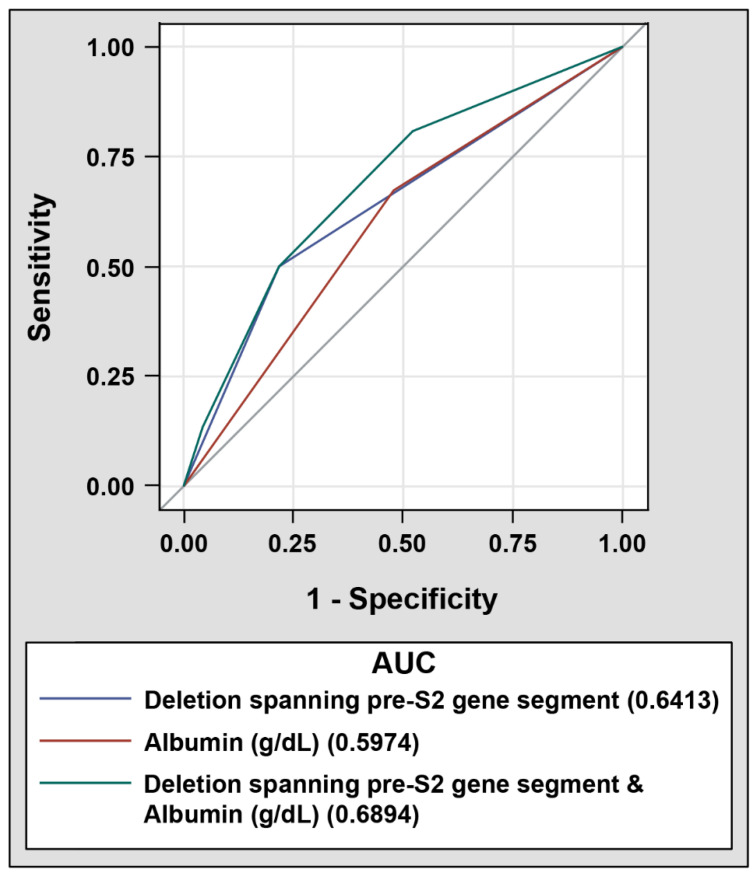
ROC curves of selected prognostic factors in discriminating patients with HCC recurrence from those without after curative surgical resection; 52 patients with and 23 patients without HCC recurrence were analyzed. (bottom) AUC for the deletion spanning the pre-S2 gene segment (solid blue line), serum albumin level (solid red line), or the combination of both factors (solid green line).

Considering the close correlation between serum albumin level and the deletion spanning the pre-S2 gene segment, the prognostic performance of combining these two factors for HCC recurrence was evaluated next. HBV-related HCC patients were divided into four groups: Group 1, absence of the deletion spanning the pre-S2 gene segment and high serum albumin level (>3.8 g/dL); Group 2, absence of the deletion spanning the pre-S2 gene segment and low serum albumin level (≤3.8 g/dL); Group 3, presence of the deletion spanning the pre-S2 gene segment and high serum albumin level; and Group 4, presence of the deletion spanning the pre-S2 gene segment and low serum albumin level. As shown in Table 5 and Figure 1C, Group 4 of patients were significantly associated with a poorer RFS than that of Group 1 of patients (median RFS, 5.9 vs. 15.6 months, *p* value = 0.0029). Moreover, ROC curve analysis revealed that a combination of the deletion spanning the pre-S2 gene segment and serum albumin level had the highest AUC (0.6894, 95% CI 0.5637 to 0.8151), followed by the deletion spanning the pre-S2 gene segment alone (0.6413, 95% CI 0.5311 to 0.7515) and serum albumin level alone (0.5974, 95% CI 0.4748 to 0.7200) (Figure 2).

## 4. Discussion

Although surgical resection is regarded as a potentially curative therapy for HCC, the high recurrence of HBV-related HCC after surgery remains a significant threat, resulting in poor patient survival [26,27]. HBV pre-S2 mutant-positive HCC patients were identified as a high-risk population for HCC recurrence after curative surgical resection, and the presence of the deletion spanning the pre-S2 gene segment in the blood is an independent biomarker for predicting HCC recurrence [21,22]. In this study, there was a significantly inverse correlation between serum albumin level and the deletion spanning the pre-S2 gene segment in HBV-related HCC patients. Furthermore, HCC patients with the presence of the deletion spanning the pre-S2 gene segment in combination with low serum albumin level displayed a higher risk of HCC recurrence than patients with either factor alone did. Our results therefore suggest that low serum albumin level is a significant clinicopathological factor, which was associated with higher HCC recurrence in pre-S2 mutant-positive HCC patients, and has promise in combination with pre-S2 mutant as a more powerful prognostic biomarker for HBV-related HCC recurrence after curative surgical resection.

Multiple studies validated the prognostic value of serum albumin level in combination with other clinicopathological factors in predicting HCC recurrence after curative surgical resection, such as the albumin–bilirubin grade combined with the fibrosis-4 index, platelet-to-lymphocyte ratio, aspartate aminotransferase-to-platelet ratio, TNM stage, or clinically significant portal hypertension [28,29,30,31,32,33]. However, the prognostic performance of serum albumin level, either alone or combined with hepatitis B viral factors, for HBV-related HCC recurrence remains poorly explored. In this study, a combination of serum albumin level and the deletion spanning the pre-S2 gene segment exhibited greater performance than that of either factor alone in identifying patients with higher risk of HBV-related HCC recurrence. Furthermore, our results reveal that pre-S2 mutant-positive HCC patients displayed significantly lower serum albumin level, and there was a close correlation between low serum albumin level and a high Child–Pugh cirrhosis score (B/C) in HBV-related HCC patients. Albumin is synthesized in the liver and is the most abundant circulating protein with multifunctional properties, such as oncotic pressure maintenance, immune modulation, endothelial stabilization, antioxidation, metabolism, and detoxification [34,35,36,37]. Low serum albumin level is a cardinal feature and prognostic biomarker of decompensated cirrhosis in patients with Child–Pugh cirrhosis scores B and C [38,39,40]. Moreover, the presence of the pre-S2 mutant in the blood is associated with a higher risk of cirrhosis development in patients with chronic HBV infection [16]. These findings therefore demonstrate that low serum albumin level in pre-S2 mutant-positive HCC patients may result from the development and progression of cirrhosis in such patients. The close association of cirrhosis with an increased risk of HCC development [41] may provide an explanation for the stronger prognostic performance of combining the serum albumin level and the deletion spanning the pre-S2 gene segment for HCC recurrence after curative surgical resection. In addition, considering that long-term albumin administration improves survival in patients with cirrhosis [42,43,44,45], such treatment may also be a promising therapeutic option for preventing HCC recurrence in pre-S2 mutant-positive HCC patients who have a low serum albumin level.

Previous research has shown that pre-S2 mutant is independently associated with late recurrence (after 1 year) but not early recurrence (within 1 year) of HCC after curative surgical resection [19], suggesting that pre-S2 mutant may be involved in de novo carcinogenesis from the precursor dysplastic lesions in the remnant liver after tumor resection rather than the dissemination of primary tumor. Consistent with this notion, among the clinicopathological factors analyzed in this study, serum albumin level was the only factor associated with pre-S2 mutant in HCC patients; however, other tumor properties such as lymph node involvement, vascular invasion, and distant metastasis showed no association with pre-S2 mutant. Whether pre-S2 mutant may promote HCC recurrence through dysregulation of serum albumin level is worth further investigation.

There are some limitations to this study. Although the causal relationships between pre-S2 mutant and cirrhosis and cirrhosis and low serum albumin level suggest that the low serum albumin level may be a result of reduced synthesis by hepatocytes in the cirrhotic liver of HCC patients with pre-S2 mutant, the elucidation of underlying molecular mechanisms would provide further insight into the development of preventive interventions for HCC recurrence in pre-S2 mutant-positive HCC patients after curative surgical resection. Furthermore, besides the decrease in serum albumin level, the molecular structure of albumin undergoes extensive damage in decompensated cirrhosis due to systemic inflammation and oxidative stress, leading to a decline in albumin functions along with the increasing severity of cirrhosis [46,47,48,49,50]. Considering that the expression of pre-S2 mutant in the liver causes chronic inflammation and oxidative stress in vitro and in vivo [12,51], it is worthwhile to assess the structural alteration of serum albumin and its prognostic value in HCC patients with the pre-S2 mutant. In addition, although the clinicopathological profile of the cohort of 75 HBV-related HCC patients enrolled in this study corresponds with representative characteristics of a large population of patients in Taiwan [52] further validation of the findings of this study in a larger cohort of patients from different clinical centers is needed. Even so, to the best of our knowledge, this study is the first to provide insights into the association between the pre-S2 mutant and clinicopathological factors, and its implications for the pathogenesis and prediction of HBV-related HCC recurrence.

## 5. Conclusions

This study provides evidence of a negative relationship between the serum albumin level and the deletion spanning the pre-S2 gene segment in HBV-related HCC patients, and validates a combination of these two factors as a potential prognostic biomarker for a higher risk of HCC recurrence after curative surgical resection.

## Figures and Tables

**Table 1:** Clinicopathological characteristics and pre-S genotyping of 75 HBV-related HCC patients enrolled in this study.

**Clinical Characteristics**	**No. of Patients**	**Median (Range)**
Age (years)	75	53 (26–78)
>50	48	60 (51–78)
≤50	27	43 (26–50)
Gender (men/women)	68/7	
Smoking (yes/no)	31/44	
Alcohol (yes/no)	29/46	
HBsAg (positive/negative/NA)	65/0/10	
HBeAg (positive/negative/NA)	9/62/4	
HBV genotype (B/C)	60/15	
HBV DNA (IU/mL) (20–1.7 × 10^8^/< 20) ^a^	74/1	2.1 × 10^4^ (21.5–1.5 × 10^8^) ^c^
>1 × 10^4^	42	4.3 × 10^5^ (1.2 × 10^4^−1.5 × 10^8^)
≤1 × 10^4^	32	8.4 × 10^2^ (21.5–9.3 × 10^3^)
Albumin (g/dL)	75	3.7 (1.2–4.9)
>3.8	30	4.2 (3.9–4.9)
≤3.8	45	3.3 (1.2–3.8)
AST (U/L)	75	60 (14–1052)
>34	61	79 (35–1052)
≤34	14	27 (14–34)
ALT (U/L)	75	55 (13–1338)
>40	50	96.5 (41–1338)
≤40	25	31 (13–40)
AFP (ng/mL) (≤54,000/>54,000) ^b^	71/4	26.7 (1.8–36,600.0) ^d^
>400	28	1920 (461.7–36,600.0)
≤400	47	13.8 (1.8–271.0)
**Pathological Characteristics**	**No. of Patients**	**Median (Range)**
Tumor size (cm)	75	4.5 (1.1–19.5)
>5	37	10.0 (5.5–19.5)
≤5	38	2.4 (1.1–4.5)
Tumor encapsulation (yes/no/NA)	42/20/13	
Lymph node involvement (yes/no)	8/67	
Portal vein thrombosis (yes/no)	5/70	
Vascular invasion (yes/no)	27/48	
Distant metastasis (yes/no)	8/67	
Steatosis grade (0/1/2/3/NA)	14/10/1/0/50	
Metavir inflammation score (0/1/2/3/NA)	4/35/5/0/31	
Ishak fibrosis score (0/1/2/3/4/5/6/NA)	5/13/12/8/3/4/11/19	
Child-Pugh cirrhosis score (A/B/C)	57/16/2	
CLIP score (0/1/2/3/4/5/6)	33/23/10/8/1/0/0	
Tumor differentiation grade (1/2/3/4)	2/36/36/1	
BCLC stage (A/B/C/D)	38/29/7/1	
AJCC TNM stage (I/II/IIIA/IIIB/IIIC/IVA/IVB)	40/20/7/5/3/0/0	
HCC recurrence after surgery (month) (yes/no)	52/23	11.2 (1.5–72.3) ^e^
**Pre-S genotyping**	**No. of Patients**	**Median (Range)**
Deletion spanning pre-S2 gene segment (presence/absence)	31/44	

^a^ HBV DNA measured with a detection range from 20 to 1.7 × 10^8^ IU/mL. ^b^ AFP measured with the highest detection limit of 54,000 ng/mL. ^c,d^ Only data within the detection range were analyzed. ^e^ Time to recurrence after surgery. Abbreviations: HBV, hepatitis B virus; HCC, hepatocellular carcinoma; HBeAg, hepatitis B e antigen; NA, not available; AST, aspartate aminotransferase; ALT, alanine aminotransferase; AFP, alpha-fetoprotein; CLIP, Cancer of the Liver Italian Program; BCLC, Barcelona Clinic Liver Cancer; AJCC, American Joint Committee on Cancer; TNM, tumor-node metastasis.

**Table 2:** Clinicopathological correlation of deletion spanning the pre-S2 gene segment in 75 HBV-related HCC patients.

**Clinical Characteristics ^a^**	**Absence (No. of Patients (%))**	**Presence (No. of Patients (%))**	***p*** **Value ^b^**
Age (years)	44 (100)	31 (100)	
>50	28 (64)	22 (71)	0.1597
≤50	16 (36)	9 (29)	
Gender	44 (100)	31 (100)	
Men	39 (89)	29 (94)	0.2544
Women	5 (11)	2 (6)	
Smoking	44 (100)	31 (100)	
Yes	18 (41)	13 (42)	0.1871
Mo	26 (59)	18 (58)	
Alcohol	44 (100)	31 (100)	
Yes	18 (41)	11 (35)	0.1710
No	26 (59)	20 (65)	
HBsAg ^c^	38 (100)	27 (100)	
Positive	38 (100)	27 (100)	
Negative	0 (0)	0 (0)	
HBeAg	42 (100)	29 (100)	
Positive	5 (12)	4 (14)	0.2713
Negative	37 (88)	25 (86)	
HBV genotype	44 (100)	31 (100)	
B	36 (82)	24 (77)	0.2044
C	8 (18)	7 (23)	
HBV DNA (copies/mL)	43 (100)	31 (100)	
>1 × 10^4^	22 (51)	20 (65)	0.0996
≤1 × 10^4^	21 (49)	11 (35)	
Albumin (g/dL)	44 (100)	31 (100)	0.0311 *
>3.8	21 (48)	8 (26)	
≤3.8	23 (52)	23 (74)	
AST (U/L)	44 (100)	31 (100)	
>34	38 (86)	23 (74)	0.0993
≤34	6 (14)	8 (26)	
ALT (U/L)	44 (100)	31 (100)	
>40	28 (64)	22 (71)	0.1597
≤40	16 (36)	9 (29)	
AFP (ng/mL)	44 (100)	31 (100)	
>400	14 (32)	14 (45)	0.0526
≤400	30 (68)	17 (55)	
**Pathological Characteristics ^a^**	**Absence (No. of Patients (%))**	**Presence (No. of Patients (%))**	***p*** **Value ^b^**
Tumor size (cm)	44 (100)	31 (100)	
>5	22 (50)	15 (48)	0.1835
≤5	22 (50)	16 (52)	
Tumor encapsulation	37 (100)	25 (100)	
yes	24 (65)	18 (72)	0.1860
no	13 (35)	7 (28)	
Lymph node involvement	44 (100)	31 (100)	
Yes	6 (14)	2 (6)	0.1946
No	38 (86)	29 (94)	
Portal vein thrombosis	44 (100)	31 (100)	
Yes	4 (9)	1 (3)	0.2438
No	40 (91)	30 (97)	
Vascular invasion	44 (100)	31 (100)	
Yes	15 (34)	12 (39)	0.1768
No	29 (66)	19 (61)	
Distant metastasis	44 (100)	31 (100)	
Yes	5 (11)	3 (10)	0.2893
No	39 (89)	28 (90)	
Steatosis grade	14 (100)	11 (100)	
2/3	1 (7)	0 (0)	0.5600
0/1	13 (93)	11 (100)	
Metavir inflammation score	24 (100)	20 (100)	
2/3	3 (13)	2 (10)	0.3541
0/1	21 (87)	18 (90)	
Ishak fibrosis score	29 (100)	27 (100)	
4/5/6	12 (41)	6 (22)	0.0723
0/1/2/3	17 (59)	21 (78)	
Child-Pugh cirrhosis score	44 (100)	31 (100)	
B/C	12 (27)	6 (19)	0.1624
A	32 (73)	25 (81)	
CLIP score	44 (100)	31 (100)	
4/5/6	0 (0)	1 (3)	0.4133
0/1/2/3	44 (100)	30 (97)	
Tumor differentiation grade	44 (100)	31 (100)	
3/4	20 (45)	17 (55)	0.1355
1/2	24 (55)	14 (45)	
BCLC stage	44 (100)	31 (100)	
C/D	6 (14)	2 (6)	0.1946
A/B	38 (86)	29 (94)	
AJCC TNM stage	44 (100)	31 (100)	
IIIA/IIIB/IIIC/IVA/IVB	8 (18)	7 (23)	0.2044
I/II	36 (82)	24 (77)	

^a^ Only patients with available data were analyzed. ^b^ *p* value determined by chi-squared test. ^c^ There were no patients negative for HBsAg for analysis. * *p* value < 0.05. Abbreviations: HBV, hepatitis B virus; HCC, hepatocellular carcinoma; HBeAg, hepatitis B e antigen; AST, aspartate aminotransferase; ALT, alanine aminotransferase; AFP, alpha-fetoprotein; CLIP, Cancer of the Liver Italian Program; BCLC, Barcelona Clinic Liver Cancer; AJCC, American Joint Committee on Cancer; TNM, tumor-node metastasis.

**Table 3:** Univariate and multivariate recurrence-free survival analyses of 75 HBV-related HCC patients.

**Clinical Characteristics**	**Univariate Analysis**			**Multivariate Analysis**		
	**HR**	**95% CI**	***p*** **Value**	**HR**	**95% CI**	***p*** **Value**
Age (years) (>50 vs. ≤50)	0.951	0.532–1.700	0.8666			
Gender (men vs. women)	1.043	0.414–2.627	0.9284			
Smoking (yes vs. no)	0.886	0.503–1.560	0.6750			
Alcohol (yes vs. no)	0.884	0.494–1.580	0.6773			
HBsAg (positive vs. negative) ^a^						
HBeAg (positive vs. negative) ^b^	1.234	0.523–2.910	0.6307			
HBV genotype (B vs. C)	0.583	0.304–1.117	0.1040			
HBV DNA (IU/mL) (>1 × 10^4^ vs. ≤1 × 10^4^) ^c^	1.645	0.934–2.895	0.0846			
Albumin (g/dL) (>3.8 vs. ≤3.8)	0.515	0.288–0.923	0.0258 *	0.658	0.349–1.241	0.1960 ^h^
AST (U/L) (>34 vs. ≤34)	0.865	0.444–1.684	0.6691			
ALT (U/L) (>40 vs. ≤40)	0.797	0.456–1.394	0.4267			
AFP (ng/mL) (>400 vs. ≤400)	1.305	0.745–2.285	0.3524			
**Pathological Characteristics**	**Univariate Analysis**			**Multivariate Analysis**		
	**HR**	**95% CI**	***p*** **Value**	**HR**	**95% CI**	***p*** **Value**
Tumor size (cm) (>5 vs. ≤5)	1.490	0.863–2.572	0.1525			
Tumor encapsulation (yes vs. no) ^d^	0.901	0.474–1.713	0.7508			
Lymph node involvement (yes vs. no)	0.333	0.104–1.071	0.0652			
Portal vein thrombosis (yes vs. no)	1.668	0.600–4.633	0.3264			
Vascular invasion (yes vs. no)	1.677	0.962–2.924	0.0681			
Distant metastasis (yes vs. no)	2.259	0.999–5.101	0.0502			
Steatosis grade (2/3 vs. 0/1) ^e^	3.473	0.418–28.879	0.2493			
Metavir inflammation score (2/3 vs. 0/1) ^f^	0.731	0.256–2.088	0.5583			
Ishak fibrosis score (4/5/6 vs. 0/1/2/3) ^g^	1.261	0.670–2.373	0.4714			
Child–Pugh cirrhosis score (B/C vs. A)	2.189	1.195–4.013	0.0112 *	1.876 2.182	0.957–3.676 1.142–4.171	0.0668 ^h^ 0.0182 *^,i^
CLIP score (4/5/6 vs. 0/1/2/3)	2.426	0.328–17.911	0.3850			
Tumor differentiation grade (3/4 vs. 1/2)	1.246	0.722–2.150	0.4288			
BCLC stage (C/D vs. A/B)	1.927	0.867–4.284	0.1077			
AJCC TNM stage (IIIA/IIIB/IIIC/IVA/IVB vs. I/II)	4.048	2.123–7.719	<0.0001 ***	3.822 3.667	1.920–7.607 1.853–7.258	0.0001 ***^,h^ 0.0002 ***^,i^
Deletion spanning pre-S2 gene segment (presence vs. absence)	1.825	1.058–3.149	0.0307 *	1.910 2.114	1.065–3.425 1.203–3.714	0.0300 *^,h^ 0.0092 **^,i^

^a^ There were no patients negative for HBsAg for analysis. ^b^ Only 71 patients with available data were analyzed. ^c^ Only 74 patients with available data were analyzed. ^d^ Only 62 patients with available data were analyzed. ^e^ Only 25 patients with available data were analyzed. ^f^ Only 44 patients with available data were analyzed. ^g^ Only 56 patients with available data were analyzed. ^h,i^ Multivariate analysis accordingly performed between these characteristics. * *p* value < 0.05; ** *p* value < 0.01; *** *p* value < 0.001. Abbreviations: HR, hazard ratio; CI, confidence interval.

**Table 4:** Clinicopathological correlation of serum albumin level in 75 HBV-related HCC patients.

**Clinical Characteristics ^a^**	**Low (No. of Patients (%))**	**High (No. of Patients (%))**	***p*** **Value ^b^**
Age (years)	46 (100)	29 (100)	
>50	29 (63)	21 (72)	0.1428
≤50	17 (37)	8 (28)	
Gender	46 (100)	29 (100)	
Men	42 (91)	26 (90)	0.3004
Women	4 (9)	3 (10)	
Smoking	46 (100)	29 (100)	
Yes	20 (43)	11 (38)	0.1710
No	26 (57)	18 (62)	
Alcohol	46 (100)	29 (100)	
Yes	19 (41)	10 (34)	0.1632
No	27 (59)	19 (66)	
HBsAg ^c^	40 (100)	25 (100)	
Positive	40 (100)	25 (100)	
Negative	0 (0)	0 (0)	
HBeAg	43 (100)	28 (100)	
Positive	6 (14)	3 (11)	0.2682
Negative	37 (86)	25 (89)	
HBV genotype	46 (100)	29 (100)	
B	36 (78)	24 (83)	0.2123
C	10 (22)	5 (17)	
HBV DNA (copies/mL)	45 (100)	29 (100)	
>1 × 10^4^	27 (60)	15 (52)	0.1487
≤1 × 10^4^	18 (40)	14 (48)	
AST (U/L)	46 (100)	29 (100)	
>34	40 (87)	21 (72)	0.0717
≤34	6 (13)	8 (28)	
ALT (U/L)	46 (100)	29 (100)	
>40	31 (67)	19 (66)	0.1949
≤40	15 (33)	10 (34)	
AFP (ng/mL)	46 (100)	29 (100)	
>400	20 (43)	8 (28)	0.0765
≤400	26 (57)	21 (72)	
**Pathological Characteristics ^a^**	**Low (No. of Patients (%))**	**High (No. of Patients (%))**	***p*** **Value ^b^**
Tumor size (cm)	46 (100)	29 (100)	
>5	26 (57)	11 (38)	0.0563
≤5	20 (43)	18 (62)	
Tumor encapsulation	35 (100)	27 (100)	
Yes	24 (69)	18 (67)	0.2124
No	11 (31)	9 (33)	
Lymph node involvement	46 (100)	29 (100)	
Yes	2 (4)	6 (21)	0.0291 *
No	44 (96)	23 (79)	
Portal vein thrombosis	46 (100)	29 (100)	
Yes	4 (9)	1 (3)	0.2742
No	42 (91)	28 (97)	
Vascular invasion	46 (100)	29 (100)	
Yes	16 (35)	11 (38)	0.1869
No	30 (65)	18 (62)	
Distant metastasis	46 (100)	29 (100)	
Yes	5 (11)	3 (10)	0.2969
No	41 (89)	26 (90)	
Steatosis grade	12 (100)	13 (100)	
2/3	0 (7)	1 (0)	0.5200
0/1	12 (93)	12 (100)	
Metavir inflammation score	26 (100)	18 (100)	
2/3	3 (12)	2 (11)	0.3663
0/1	23 (88)	16 (89)	
Ishak fibrosis score	35 (100)	21 (100)	
4/5/6	11 (31)	7 (33)	0.2285
0/1/2/3	24 (69)	14 (67)	
Child–Pugh cirrhosis score	46 (100)	29 (100)	
B/C	16 (35)	2 (7)	0.0042 **
A	30 (65)	27 (93)	
CLIP score	46 (100)	29 (100)	
4/5/6	0 (0)	1 (3)	0.3867
0/1/2/3	46 (100)	28 (97)	
Tumor differentiation grade	46 (100)	29 (100)	
3/4	24 (52)	13 (45)	0.1554
1/2	22 (48)	16 (55)	
BCLC stage	46 (100)	29 (100)	
C/D	6 (13)	2 (7)	0.2254
A/B	40 (87)	27 (93)	
AJCC TNM stage	46 (100)	29 (100)	
IIIA/IIIB/IIIC/IVA/IVB	11 (24)	4 (14)	0.1390
I/II	35 (76)	25 (86)	

^a^ Only patients with available data were analyzed. ^b^ *p* value was determined by chi-squared test. ^c^ There were no patients negative for HBsAg for analysis. * *p* value < 0.05; ** *p* value < 0.01. Abbreviations: HBV, hepatitis B virus; HCC, hepatocellular carcinoma; HBeAg, hepatitis B e antigen; AST, aspartate aminotransferase; ALT, alanine aminotransferase; AFP, alpha-fetoprotein; CLIP, Cancer of the Liver Italian Program; BCLC, Barcelona Clinic Liver Cancer; AJCC, American Joint Committee on Cancer; TNM, tumor-node metastasis.

**Table 5:** Univariate recurrence-free survival analysis of patients with deletion spanning the pre-S2 gene segment and/or albumin.

Characteristics	HR	95% CI	*p* Value
Deletion spanning pre-S2 gene segment (presence vs. absence)	1.825	1.058–3.149	0.0307 *
Albumin (g/dL) (>3.8 vs. ≤3.8)	0.515	0.288–0.923	0.0258 *
Deletion spanning pre-S2 gene segment and Albumin (g/dL) (absence and ≤3.8 vs. absence and >3.8)	1.835	0.831–4.053	0.1333
Deletion spanning pre-S2 gene segment and Albumin (g/dL) (presence and >3.8 vs. absence and >3.8)	1.722	0.654–4.531	0.2709
Deletion spanning pre-S2 gene segment and Albumin (g/dL) (presence and ≤3.8 vs. absence and >3.8)	3.087	1.427–6.679	0.0042 **

* *p* value < 0.05; ** *p* value < 0.01. Abbreviations: HR, hazard ratio; CI, confidence interval.

## Data Availability

All data generated or analyzed during this study are included in this published article and its supplementary materials.

## Supplementary Materials

The following are available online at https://www.mdpi.com/article/10.3390/jcm10184187/s1, Table S1. List of NGS-based pre-S genotyping result in 75 HBV-related HCC patients.
