# Supplementary material for: Association of Low Serum Albumin Level with Higher Hepatocellular Carcinoma Recurrence in Patients with Hepatitis B Virus Pre-S2 Mutant after Curative Surgical Resection
Source: J Clin Med. 2021 Sep 16;10(18):4187. doi: 10.3390/jcm10184187 (PMC8464848; doi:10.3390/jcm10184187)
Supplement: Supplementary file 1 [file jcm-10-04187-s001.zip › jcm-1372733-supplementary.pdf]

Table S1. List of the NGS-based pre-S genotyping result in 75 HBV-related HCC patients

Patient No.	Pre-S Del Type (%)[a]	Pre-S Del Region (%)[c]	Del Spanning Pre-S2 Gene Segment[d]
1	1. **pre-S1 del (92.118)**[b]	1. pre-S1 del (nt 2855-2872) (90.102)	absence
	**2. wild-type (7.278)**	2. wild-type (7.278)	
	3. pre-S2 del (0.372)	3. pre-S2 del (nt 1-28) (0.180)	
	4. pre-S1+pre-S2 del (0.231)	4. pre-S1+pre-S2 del (nt 2855-2872, 1-111) (0.027)	
2	1. **pre-S1 del (75.241)**	1. pre-S1 del (nt 2910-3089) (48.977)	absence
	2. **wild-type (22.338)**	2. wild-type (22.338)	
	3. pre-S2 del (1.891)	3. pre-S2 del (nt 1-54) (1.579)	
	4. pre-S1+pre-S2 del (0.530)	4. pre-S1+pre-S2 del (nt 2910-3089, 1-57) (0.277)	
3	1. **pre-S1 del (76.228)**	1. pre-S1 del (nt 3110-3127) (24.869)	presence
	2. **wild-type (12.583)**	2. wild-type (12.583)	
	3. **pre-S2 del (10.622)**	3. pre-S2 del (nt 1-57) (10.000)	
	4. pre-S1+pre-S2 del (0.567)	4. pre-S1+pre-S2 del (nt 2855-2872, 1-57) (0.490)	
4	1. **wild-type (71.590)**	1. wild-type (71.590)	absence
	2. **pre-S1 del (28.270)**	2. pre-S1 del (nt 2854-3147) (23.961)	
	3. pre-S2 del (0.129)	3. pre-S2 del (nt 3211-3216) (0.048)	
	4. pre-S1+pre-S2 del (0.011)	4. pre-S1+pre-S2 del (nt 2954-3097, 1-54) (0.002)	
5	1. **wild-type (58.461)**	1. wild-type (58.461)	presence
	2. **pre-S2 del (37.194)**	2. pre-S2 del (nt 24-50) (33.801)	
	3. pre-S1 del (2.939)	3. pre-S1 del (nt 2880-3146) (0.646)	
	4. pre-S1+pre-S2 del (1.407)	4. pre-S1+pre-S2 del (nt 2880-3146, 24-50) (0.355)	
6	1. **wild-type (96.592)**	1. wild-type (96.592)	absence
	2. pre-S1 del (2.906)	2. pre-S1 del (nt 3026-3205) (0.604)	
	3. pre-S2 del (0.469)	3. pre-S2 del (nt 1-12) (0.233)	
	4. pre-S1+pre-S2 del (0.033)	4. pre-S1+pre-S2 del (nt 2854-2979, 6-134) (0.008)	

Table S1. List of the NGS-based pre-S genotyping result in 75 HBV-related HCC patients (continued)

Patient No.	Pre-S Del Type (%)[a]	Pre-S Del Region (%)[c]	Del Spanning Pre-S2 Gene Segment[d]
7	1. **wild-type (99.000)**	1. wild-type (99.000)	absence
	2. pre-S1 del (0.927)	2. pre-S1 del (nt 3106-3129) (0.294)	
	3. pre-S2 del (0.062)	3. pre-S2 del (nt 1-54) (0.031)	
	4. pre-S1+pre-S2 del (0.012)	4. pre-S1+pre-S2 del (nt 2855-2972, 1-54) (0.008)	
8	1. **wild-type (93.746)**	1. wild-type (93.746)	absence
	2. **pre-S1 del (5.846)**	2. pre-S1 del (nt 3026-3205) (2.417)	
	3. pre-S2 del (0.389)	3. pre-S2 del (nt 3211-3213) (0.135)	
	4. pre-S1+pre-S2 del (0.018)	4. pre-S1+pre-S2 del (nt 2856-2873, 1-21) (0.004)	
9	1. **pre-S1+pre-S2 del (46.237)**	1. pre-S1+pre-S2 del (nt 2956-3126, 1-9) (24.002)	presence
	2. **pre-S2 del (26.927)**	2. pre-S2 del (nt 1-54) (23.226)	
	3. **pre-S1 del (14.368)**	3. pre-S1 del (nt 2944-3075) (5.140)	
	4. **wild-type (12.467)**	4. wild-type (12.467)	
10	1. **wild-type (97.851)**	1. wild-type (97.851)	absence
	2. pre-S1 del (2.037)	2. pre-S1 del (nt 3026-3205) (1.109)	
	3. pre-S2 del (0.108)	3. pre-S2 del (nt 3211-3213) (0.049)	
	4. pre-S1+pre-S2 del (0.005)	4. pre-S1+pre-S2 del (nt 2855-2872, 1-54) (0.003)	
11	1. **wild-type (98.964)**	1. wild-type (98.964)	absence
	2. pre-S1 del (0.943)	2. pre-S1 del (nt 2854-2970) (0.125)	
	3. pre-S2 del (0.084)	3. pre-S2 del (nt 1-9) (0.028)	
	4. pre-S1+pre-S2 del (0.009)	4. pre-S1+pre-S2 del (nt 2954-3097, 1-13) (0.009)	
12	1. **wild-type (97.781)**	1. wild-type (97.781)	absence
	2. pre-S1 del (2.099)	2. pre-S1 del (nt 3026-3205) (1.099)	
	3. pre-S2 del (0.112)	3. pre-S2 del (nt 3211-3216) (0.055)	
	4. pre-S1+pre-S2 del (0.008)	4. pre-S1+pre-S2 del (nt 3026-3205, 43-138) (0.002)	

Table S1. List of the NGS-based pre-S genotyping result in 75 HBV-related HCC patients (continued)

Patient No.	Pre-S Del Type (%)[a]	Pre-S Del Region (%)[c]	Del Spanning Pre-S2 Gene Segment[d]
13	1. **wild-type (92.291)**	1. wild-type (92.291)	absence
	2. pre-S1 del (4.092)	2. pre-S1 del (nt 2854-2970) (0.650)	
	3. pre-S2 del (3.038)	3. pre-S2 del (nt 1-54) (2.641)	
	4. pre-S1+pre-S2 del (0.578)	4. pre-S1+pre-S2 del (nt 2855-2872, 1-54) (0.435)	
14	1. **wild-type (69.001)**	1. wild-type (69.001)	presence
	2. **pre-S1 del (20.530)**	2. pre-S1 del (nt 3110-3127) (4.779)	
	3**. pre-S2 del (9.463)**	3. pre-S2 del (nt 1-54) (8.226)	
	4. pre-S1+pre-S2 del (1.006)	4. pre-S1+pre-S2 del (nt 2855-2872, 1-18, 26-59) (0.259)	
15	1. **wild-type (50.938)**	1. wild-type (50.938)	presence
	2. **pre-S1 del (19.760)**	2. pre-S1 del (nt 2854-2970) (5.470)	
	3. **pre-S1+pre-S2 del (15.021)**	3. pre-S1+pre-S2 del (nt 2855-2872, 1-54) (12.421)	
	4. **pre-S2 del (14.280)**	4. pre-S2 del (nt 1-54) (13.606)	
16	1. **wild-type (75.230)**	1. wild-type (75.230)	presence
	2. **pre-S1+pre-S2 del (13.878)**	2. pre-S1+pre-S2 del (nt 2860-2880, 2954-3097, 1-15) (11.572)	
	3. **pre-S1 del (10.730)**	3. pre-S1 del (nt 2860-2880, 2954-3097) (9.550)	
	4. pre-S2 del (0.162)	4. pre-S2 del (nt 1-15) (0.138)	
17	1. **wild-type (98.571)**	1. wild-type (98.571)	absence
	2. pre-S1 del (1.173)	2. pre-S1 del (nt 3106-3129) (0.242)	
	3. pre-S2 del (0.178)	3. pre-S2 del (nt 2-149) (0.043)	
	4. pre-S1+pre-S2 del (0.078)	4. pre-S1+pre-S2 del (nt 2854-2985, 27-149) (0.014)	
18	1. **wild-type (96.915)**	1. wild-type (96.915)	absence
	2. pre-S1 del (2.701)	2. pre-S1 del (nt 3026-3205) (1.755)	
	3. pre-S2 del (0.364)	3. pre-S2 del (nt 25-54) (0.148)	
	4. pre-S1+pre-S2 del (0.020)	4. pre-S1+pre-S2 del (nt 2854-2988, 45-149) (0.004)	

Table S1. List of the NGS-based pre-S genotyping result in 75 HBV-related HCC patients (continued)

Patient No.	Pre-S Del Type (%)[a]	Pre-S Del Region (%)[c]	Del Spanning Pre-S2 Gene Segment[d]
19	1. **wild-type (98.069)**	1. wild-type (98.069)	absence
	2. pre-S1 del (1.263)	2. pre-S1 del (nt 3103-3126) (0.253)	
	3. pre-S2 del (0.609)	3. pre-S2 del (nt 1-15) (0.535)	
	4. pre-S1+pre-S2 del (0.059)	4. pre-S1+pre-S2 del (nt 2854-2996, 44-144) (0.015)	
20	1. **wild-type (69.571)**	1. wild-type (69.571)	presence
	2. **pre-S2 del (18.590)**	2. pre-S2 del (nt 1-54) (18.482)	
	**3. pre-S1+pre-S2 del (8.224)**	3. pre-S1+pre-S2 del (nt 2855-2872, 1-54) (7.712)	
	4. pre-S1 del (3.615)	4. pre-S1 del (nt 2855-2872) (2.674)	
21	1. **pre-S2 del (41.477)**	1. pre-S2 del (nt 1-54) (24.620)	presence
	2. **pre-S1+pre-S2 del (39.126)**	2. pre-S1+pre-S2 del (nt 2855-2872, 1-54) (26.451)	
	3. **wild-type (12.348)**	3. wild-type (12.348)	
	4. **pre-S1 del (7.048)**	4. pre-S1 del (nt 2855-2872) (4.978)	
22	1. **pre-S2 del (42.909)**	1. pre-S2 del (nt 1-54) (38.091)	presence
	2. **pre-S1+pre-S2 del (27.915)**	2. pre-S1+pre-S2 del (2855-2872, 1-54) (25.090)	
	3. **wild-type (17.564)**	3. wild-type (17.564)	
	4. **pre-S1 del (11.612)**	4. pre-S1 del (nt 2855-2872) (8.072)	
23	1. **wild-type (52.661)**	1. wild-type (52.661)	presence
	2. **pre-S2 del (25.093)**	2. pre-S2 del (nt 1-54) (18.682)	
	3. **pre-S1+pre-S2 del (11.424)**	3. pre-S1+pre-S2 del (nt 2855-2872, 1-54) (8.344)	
	4. **pre-S1 del (10.821)**	4. pre-S1 del (nt 2855-2872) (2.817)	
24	1. **wild-type (96.073)**	1. wild-type (96.073)	absence
	2. pre-S1 del (1.895)	2. pre-S1 del (nt 2954-3097) (0.632)	
	3. pre-S1+pre-S2 del (1.202)	3. pre-S1+pre-S2 del (nt 2855-2872, 1-54) (0.452)	
	4. pre-S2 del (0.830)	4. pre-S2 del (nt 1-54) (0.382)	

Table S1. List of the NGS-based pre-S genotyping result in 75 HBV-related HCC patients (continued)

Patient No.	Pre-S Del Type (%)[a]	Pre-S Del Region (%)[c]	Del Spanning Pre-S2 Gene Segment[d]
25	1. **wild-type (93.547)**	1. wild-type (93.547)	absence
	2. pre-S1 del (4.345)	2. pre-S1 del (nt 3026-3205) (0.869)	
	3. pre-S2 del (1.844)	3. pre-S2 del (nt 1-54) (1.107)	
	4. pre-S1+pre-S2 del (0.265)	4. pre-S1+pre-S2 del (nt 2855-2872, 2897-2923, 1-54) (0.180)	
26	1. **wild-type (97.921)**	1. wild-type (97.921)	absence
	2. pre-S1 del (1.388)	2. pre-S1 del (nt 3067-3162) (0.040)	
	3. pre-S2 del (0.691)	3. pre-S2 del (nt 3211-3216) (0.046)	
	4. pre-S1+pre-S2 del (0.000)	4. pre-S1+pre-S2 del (0.000)	
27	1. **wild-type (60.195)**	1. wild-type (60.195)	presence
	2. **pre-S2 del (22.882)**	2. pre-S2 del (nt 1-57) (22.473)	
	3. **pre-S1+pre-S2 del (9.280)**	3. pre-S1+pre-S2 del (nt 2855-2872, 1-54) (4.815)	
	4. **pre-S1 del (7.643)**	4. pre-S1 del (nt 3025-3126) (4.396)	
28	1. **pre-S1 del (75.113)**	1. pre-S1 del (nt 2855-2872) (5.979)	presence
	2. **wild-type (11.506)**	2. wild-type (11.506)	
	3. **pre-S1+pre-S2 del (10.472)**	3. pre-S1+pre-S2 del (nt 2855-2872, 1-54) (8.016)	
	4. pre-S2 del (2.909)	4. pre-S2 del (nt 1-54) (2.747)	
29	1. **wild-type (97.730)**	1. wild-type (97.730)	absence
	2. pre-S1 del (1.691)	2. pre-S1 del (nt 2855-2970) (0.217)	
	3. pre-S2 del (0.575)	3. pre-S2 del (nt 1-12) (0.323)	
	4. pre-S1+pre-S2 del (0.004)	4. pre-S1+pre-S2 del (nt 2855-2970, 1-54) (0.004)	
30	1. **wild-type (87.018)**	1. wild-type (87.018)	absence
	2. **pre-S1 del (11.703)**	2. pre-S1 del (nt 3010-3075) (5.189)	
	3. pre-S2 del (1.113)	3. pre-S2 del (nt 1-9) (0.392)	
	4. pre-S1+pre-S2 del (0.166)	4. pre-S1+pre-S2 del (nt 3010-3075, 1-9) (0.046)	

Table S1. List of the NGS-based pre-S genotyping result in 75 HBV-related HCC patients (continued)

Patient No.	Pre-S Del Type (%)[a]	Pre-S Del Region (%)[c]	Del Spanning Pre-S2 Gene Segment[d]
31	1. **wild-type (78.709)**	1. wild-type (78.709)	absence
	2. **pre-S1 deletion (20.616)**	2. pre-S1 del (nt 2910-3089) (9.181)	
	3. pre-S2 del (0.646)	3. pre-S2 del (nt 1-15) (0.345)	
	4. pre-S1+pre-S2 del (0.029)	4. pre-S1+pre-S2 del (nt 2910-3055, 3067-3089, 4-12) (0.007)	
32	1. **wild-type (98.105)**	1. wild-type (98.105)	absence
	2. pre-S1 del (1.152)	2. pre-S1 del (nt 2855-2970) (0.060)	
	3. pre-S2 del (0.733)	3. pre-S2 del (nt 1-9) (0.408)	
	4. pre-S1+pre-S2 del (0.010)	4. pre-S1+pre-S2 del (nt 2855-2872, 1-54) (0.003)	
33	1. **wild-type (80.067)**	1. wild-type (80.067)	presence
	2. **pre-S2 del (9.477)**	2. pre-S2 del (nt 1-12) (8.957)	
	3. **pre-S1 del (8.993)**	3. pre-S1 del (nt 2866-3075) (7.322)	
	4. pre-S1+pre-S2 del (1.463)	4. pre-S1+pre-S2 del (nt 2866-3075, 1-9) (0.871)	
34	1. **wild-type (80.175)**	1. wild-type (80.175)	presence
	2. **pre-S2 del (13.639)**	2. pre-S2 del (nt 1-30) (4.394)	
	3. **pre-S1 del (5.169)**	3. pre-S1 del (nt 2865-2975) (2.356)	
	4. pre-S1+pre-S2 del (1.017)	4. pre-S1+pre-S2 del (nt 2866-2975, 1-29) (0.212)	
35	1. **wild-type (92.156)**	1. wild-type (92.156)	absence
	2. **pre-S1 del (7.022)**	2. pre-S1 del (nt 2854-3018) (1.346)	
	3. pre-S2 del (0.793)	3. pre-S2 del (nt 1-9) (0.323)	
	4. pre-S1+pre-S2 del (0.029)	4. pre-S1+pre-S2 del (nt 2855-2872, 1-9) (0.004)	
36	1. **pre-S1+pre-S2 del (40.433)**	1. pre-S1+pre-S2 del (nt 2855-2872, 1-54) (22.713)	presence
	2. **pre-S1 del (34.174)**	2. pre-S1 del (nt 2854-2970) (22.956)	
	3. **wild-type (20.822)**	3. wild-type (20.822)	
	4. pre-S2 del (4.571)	4. pre-S2 del (nt 1-54) (4.380)	

Table S1. List of the NGS-based pre-S genotyping result in 75 HBV-related HCC patients (continued)

Patient No.	Pre-S Del Type (%)[a]	Pre-S Del Region (%)[c]	Del Spanning Pre-S2 Gene Segment[d]
37	1. **wild-type (80.758)**	1. wild-type (80.758)	presence
	2. **pre-S2 del (12.910)**	2. pre-S2 del (nt 1-54) (6.944)	
	3. **pre-S1 del (5.629)**	3. pre-S1 del (nt 2855-2875) (4.209)	
	4. pre-S1+pre-S2 del (0.704)	4. pre-S1+pre-S2 del (nt 2855-2875, 1-54) (0.253)	
38	1. **wild-type (61.320)**	1. wild-type (61.320)	absence
	2. **pre-S1 del (34.045)**	2. pre-S1 del (nt 3021-3203) (23.818)	
	3. pre-S2 del (3.491)	3. pre-S2 del (nt 6-41) (1.385)	
	4. pre-S1+pre-S2 del (1.144)	4. pre-S1+pre-S2 del (nt 2855-2872, 2919-3126, 6-41) (0.163)	
39	1. **wild-type (97.172)**	1. wild-type (97.172)	absence
	2. pre-S1 del (1.957)	2. pre-S1 del (nt 3021-3203) (0.353)	
	3. pre-S2 del (0.850)	3. pre-S2 del (nt 1-12) (0.488)	
	4. pre-S1+pre-S2 del (0.021)	4. pre-S1+pre-S2 del (nt 3078-3094, 6-41) (0.003)	
40	1. **wild-type (97.501)**	1. wild-type (97.501)	absence
	2. pre-S1 del (1.586)	2. pre-S1 del (nt 3107-3202) (0.081)	
	3. pre-S2 del (0.908)	3. pre-S2 del (nt 1-12) (0.529)	
	4. pre-S1+pre-S2 del (0.005)	4. pre-S1+pre-S2 del (nt 3133-3136, 1-10) (0.005)	
41	1. **pre-S1 del (84.262)**	1. pre-S1 del (nt 2858-2986) (82.700)	absence
	2. **wild-type (14.798)**	2. wild-type (14.798)	
	3. pre-S1+pre-S2 del (0.779)	3. pre-S1+pre-S2 del (nt 2858-2981, 1-10) (0.268)	
	4. pre-S2 del (0.161)	4. pre-S2 del (nt 1-33) (0.053)	
42	1. **wild-type (97.757)**	1. wild-type (97.757)	absence
	2. pre-S1 del (1.305)	2. pre-S1 del (nt 3106-3207) (0.060)	
	3. pre-S2 del (0.923)	3. pre-S2 del (nt 3211-3213) (0.124)	
	4. pre-S1+pre-S2 del (0.015)	4. pre-S1+pre-S2 del (nt 3069-3118, 3211-3212) (0.004)	

Table S1. List of the NGS-based pre-S genotyping result in 75 HBV-related HCC patients (continued)

Patient No.	Pre-S Del Type (%)[a]	Pre-S Del Region (%)[c]	Del Spanning Pre-S2 Gene Segment[d]
43	1. **pre-S2 del (49.695)**	1. pre-S2 del (nt 37-54) (43.929)	presence
	2. **wild-type (49.248)**	2. wild-type (49.248)	
	3. pre-S1+pre-S2 del (0.814)	3. pre-S1+pre-S2 del (nt 3139-3142, 37-54) (0.049)	
	4. pre-S1 del (0.242)	4. pre-S1 del (nt 3103-3200) (0.019)	
44	1. **wild-type (98.244)**	1. wild-type (98.244)	absence
	2. pre-S1 del (1.113)	2. pre-S1 del (nt 3089-3202) (0.049)	
	3. pre-S2 del (0.643)	3. pre-S2 del (nt 1-15) (0.319)	
	4. pre-S1+pre-S2 del (0.000)	4. pre-S1+pre-S2 del (0.000)	
45	1. **wild-type (97.534)**	1. wild-type (97.534)	absence
	2. pre-S1 del (1.376)	2. pre-S1 del (nt 3106-3207) (0.063)	
	3. pre-S2 del (1.076)	3. pre-S2 del (nt 1-9) (0.769)	
	4. pre-S1+pre-S2 del (0.014)	4. pre-S1+pre-S2 del (nt 3095-3150, 1-12) (0.007)	
46	1. **wild-type (97.735)**	1. wild-type (97.735)	absence
	2. pre-S1 del (1.534)	2. pre-S1 del (nt 3138-3197) (0.111)	
	3. pre-S2 del (0.731)	3. pre-S2 del (nt 1-9) (0.371)	
	4. pre-S1+pre-S2 del (0.000)	4. pre-S1+pre-S2 del (0.000)	
47	1. **wild-type (78.334)**	1. wild-type (78.334)	absence
	2. **pre-S1 del (20.695)**	2. pre-S1 del (nt 2895-3140) (15.887)	
	3. pre-S2 del (0.866)	3. pre-S2 del (nt 1-9) (0.279)	
	4. pre-S1+pre-S2 del (0.105)	4. pre-S1+pre-S2 del (nt 2895-3141, 1-19, 23-27) (0.023)	
48	1. **pre-S1 del (57.159)**	1. pre-S1 del (nt 2968-3093) (47.067)	presence
	2. **wild-type (20.456)**	2. wild-type (20.456)	
	3. **pre-S2 del (13.666)**	3. pre-S2 del (nt 1-54) (8.120)	
	4. **pre-S1+pre-S2 del (8.719)**	4. pre-S1+pre-S2 del (nt 2968-3093, 1-54) (3.469)	

Table S1. List of the NGS-based pre-S genotyping result in 75 HBV-related HCC patients (continued)

Patient No.	Pre-S Del Type (%)[a]	Pre-S Del Region (%)[c]	Del Spanning Pre-S2 Gene Segment[d]
49	1. **wild-type (75.021)**	1. wild-type (75.021)	presence
	2. **pre-S2 del (21.983)**	2. pre-S2 del (nt 1-1) (19.454)	
	3. pre-S1 del (2.327)	3. pre-S1 del (nt 2855-2872) (0.704)	
	4. pre-S1+pre-S2 del (0.669)	4. pre-S1+pre-S2 del (nt 2855-2872, 1-54) (0.606)	
50	1. **wild-type (98.169)**	1. wild-type (98.169)	absence
	2. pre-S1 del (1.138)	2. pre-S1 del (nt 3138-3197) (0.067)	
	3. pre-S2 del (0.693)	3. pre-S2 del (nt 1-9) (0.377)	
	4. pre-S1+pre-S2 del (0.000)	4. pre-S1+pre-S2 del (0.000)	
51	1. **wild-type (97.734)**	1. wild-type (97.734)	absence
	2. pre-S1 del (1.546)	2. pre-S1 del (nt 3104-3202) (0.165)	
	3. pre-S2 del (0.719)	3. pre-S2 del (nt 1-9) (0.377)	
	4. pre-S1+pre-S2 del (0.000)	4. pre-S1+pre-S2 del (0.000)	
52	1. **wild-type (98.167)**	1. wild-type (98.167)	absence
	2. pre-S1 del (1.192)	2. pre-S1 del (nt 2854-3128) (0.132)	
	3. pre-S2 del (0.617)	3. pre-S2 del (nt 1-9) (0.246)	
	4. pre-S1+pre-S2 del (0.024)	4. pre-S1+pre-S2 del (nt 3022-3125, 1-55) (0.018)	
53	1. **wild-type (97.002)**	1. wild-type (97.002)	absence
	2. pre-S1 del (2.144)	2. pre-S1 del (nt 3026-3205) (0.927)	
	3. pre-S2 del (0.849)	3. pre-S2 del (nt 1-9) (0.528)	
	4. pre-S1+pre-S2 del (0.005)	4. pre-S1+pre-S2 del (nt 3020-3119, 1-55) (0.005)	
54	1. **wild-type (96.687)**	1. wild-type (96.687)	absence
	2. pre-S1 del (2.625)	2. pre-S1 del (nt 2855-2872) (1.117)	
	3. pre-S2 del (0.676)	3. pre-S2 del (nt 1-12) (0.411)	
	4. pre-S1+pre-S2 del (0.013)	4. pre-S1+pre-S2 del (nt 2855-2872, 1-17) (0.004)	

Table S1. List of the NGS-based pre-S genotyping result in 75 HBV-related HCC patients (continued)

Patient No.	Pre-S Del Type (%)[a]	Pre-S Del Region (%)[c]	Del Spanning Pre-S2 Gene Segment[d]
55	1. **wild-type (64.666)**	1. wild-type (64.666)	absence
	2. **pre-S1 del (34.582)**	2. pre-S1 del (nt 2858-2986) (33.466)	
	3. pre-S2 del (0.493)	3. pre-S2 del (nt 1-9) (0.303)	
	4. pre-S1+pre-S2 del (0.259)	4. pre-S1+pre-S2 del (nt 2858-2981, 1-10) (0.069)	
56	1. **pre-S1 del (69.372)**	1. pre-S1 del (nt 2856-3101) (66.854)	absence
	2. **wild-type (30.404)**	2. wild-type (30.404)	
	3. pre-S2 del (0.218)	3. pre-S2 del (nt 1-12) (0.130)	
	4. pre-S1+pre-S2 del (0.005)	4. pre-S1+pre-S2 del (nt 2984-3098, 1-10) (0.002)	
57	1. **wild-type (94.005)**	1. wild-type (94.005)	absence
	2. pre-S1 del (3.729)	2. pre-S1 del (nt 2895-3188) (0.677)	
	3. pre-S2 del (1.824)	3. pre-S2 del (nt 1-54) (1.029)	
	4. pre-S1+pre-S2 del (0.442)	4. pre-S1+pre-S2 del (nt 2855-2872, 1-54) (0.253)	
58	1. **wild-type (95.575)**	1. wild-type (95.575)	absence
	2. pre-S1 del (2.481)	2. pre-S1 del (nt 3039-3092) (0.590)	
	3. pre-S2 del (1.931)	3. pre-S2 del (nt 1-12) (0.510)	
	4. pre-S1+pre-S2 del (0.013)	4. pre-S1+pre-S2 del (nt 2854-2940, 1-144) (0.007)	
59	1. **pre-S2 del (64.182)**	1. pre-S2 del (nt 15-56) (33.613)	presence
	2. **wild-type (32.367)**	2. wild-type (32.367)	
	3. pre-S1+pre-S2 del (2.975)	3. pre-S1+pre-S2 del (nt 3088-3126, 15-56) (0.176)	
	4. pre-S1 del (0.476)	4. pre-S1 del (nt 2923-3090) (0.073)	
60	1. **pre-S1 del (38.192)**	1. pre-S1 del (nt 2854-2970) (27.998)	presence
	2. **wild-type (34.644)**	2. wild-type (30.404)	
	3. **pre-S2 del (19.195)**	3. pre-S2 del (nt 1-54) (18.373)	
	4. **pre-S1+pre-S2 del (7.968)**	4. pre-S1+pre-S2 del (nt 3025-3126, 1-57) (2.383)	

Table S1. List of the NGS-based pre-S genotyping result in 75 HBV-related HCC patients (continued)

Patient No.	Pre-S Del Type (%)[a]	Pre-S Del Region (%)[c]	Del Spanning Pre-S2 Gene Segment[d]
61	1. **pre-S1 del (40.086)**	1. pre-S1 del (nt 2855-2965) (24.125)	presence
	2. **wild-type (34.919)**	2. wild-type (34.919)	
	3. **pre-S2 del (16.065)**	3. pre-S2 del (nt 1-54) (15.565)	
	4. **pre-S1+pre-S2 del (8.930)**	4. pre-S1+pre-S2 del (nt 3022-3126, 1-60) (2.911)	
62	1. **pre-S1 del (29.181)**	1. pre-S1 del (nt 2856-2969) (17.015)	presence
	2. **wild-type (23.836)**	2. wild-type (23.836)	
	3. **pre-S2 del (23.645)**	3. pre-S2 del (nt 1-54) (23.402)	
	4. **pre-S1+pre-S2 del (23.338)**	4. pre-S1+pre-S2 del (nt 2855-2872, 1-54) (15.956)	
63	1. **pre-S1 del (52.401)**	1. pre-S1 del (nt 2854-2970) (43.037)	absence
	2. **wild-type (42.979)**	2. wild-type (42.979)	
	3. pre-S2 del (2.312)	3. pre-S2 del (nt 1-54) (1.977)	
	4. pre-S1+pre-S2 del (2.308)	4. pre-S1+pre-S2 del (nt 2860-2880, 2954-3097, 1-15) (1.445)	
64	1. **pre-S1 del (41.155)**	1. pre-S1 del (nt 2854-2970) (35.773)	presence
	2. **wild-type (37.573)**	2. wild-type (37.573)	
	3. **pre-S2 del (18.905)**	3. pre-S2 del (nt 1-54) (18.494)	
	4. pre-S1+pre-S2 del (2.367)	4. pre-S1+pre-S2 del (nt 2855-2970, 1-54) (1.676)	
65	1. **pre-S1 del (63.334)**	1. pre-S1 del (nt 2854-2970) (33.450)	absence
	2. **wild-type (32.806)**	2. wild-type (32.806)	
	3. pre-S2 del (1.965)	3. pre-S2 del (nt 1-54) (1.708)	
	4. pre-S1+pre-S2 del (1.895)	4. pre-S1+pre-S2 del (nt 2855-2970, 1-54) (0.994)	
66	1. **wild-type (30.973)**	1. wild-type (30.973)	presence
	2. **pre-S1+pre-S2 del (27.774)**	2. pre-S1+pre-S2 del (nt 2855-2872, 1-54) (24.398)	
	3. **pre-S1 del (27.161)**	3. pre-S1 del (nt 2854-2970) (14.813)	
	4. **pre-S2 del (14.091)**	4. pre-S2 del (nt 1-54) (13.846)	

Table S1. List of the NGS-based pre-S genotyping result in 75 HBV-related HCC patients (continued)

Patient No.	Pre-S Del Type (%)[a]	Pre-S Del Region (%)[c]	Del Spanning Pre-S2 Gene Segment[d]
67	1. **wild-type (46.914)**	1. wild-type (46.914)	presence
	2. **pre-S1 del (45.517)**	2. pre-S1 del (nt 2854-2970) (37.666)	
	3. **pre-S2 del (6.834)**	3. pre-S2 del (nt 1-54) (5.216)	
	4. pre-S1+pre-S2 del (0.735)	4. pre-S1+pre-S2 del (nt 2854-2970, 1-54) (0.307)	
68	1. **pre-S1 del (43.130)**	1. pre-S1 del (nt 2854-2970) (35.888)	presence
	2. **wild-type (41.965)**	2. wild-type (41.965)	
	3. **pre-S2 del (9.508)**	3. pre-S2 del (nt 1-54) (9.239)	
	4. **pre-S1+pre-S2 del (5.397)**	4. pre-S1+pre-S2 del (nt 2855-2872, 1-54) (4.160)	
69	1. **wild-type (36.868)**	1. wild-type (36.868)	presence
	2. **pre-S1 del (35.238)**	2. pre-S1 del (nt 2854-2970) (29.499)	
	3. **pre-S2 del (21.130)**	3. pre-S2 del (nt 1-54) (20.218)	
	4. **pre-S1+pre-S2 del (6.763)**	4. pre-S1+pre-S2 del (nt 2855-2872, 1-54) (3.332)	
70	1. **wild-type (94.788)**	1. wild-type (94.788)	absence
	2. **pre-S1 del (4.643)**	2. pre-S1 del (nt 2854-3021) (3.404)	
	3. pre-S2 del (0.455)	3. pre-S2 del (nt 1-15) (0.192)	
	4. pre-S1+pre-S2 del (0.114)	4. pre-S1+pre-S2 del (nt 2855-2872, 1-54) (0.086)	
71	1. **pre-S2 del (54.981)**	1. pre-S2 del (nt 48-56) (52.982)	presence
	2. **wild-type (42.238)**	2. wild-type (42.238)	
	3. pre-S1+pre-S2 del (2.274)	3. pre-S1+pre-S2 del (nt 3026-3205, 48-56) (0.916)	
	4. pre-S1 del (0.507)	4. pre-S1 del (nt 2854-3021) (0.334)	
72	1. **wild-type (91.511)**	1. wild-type (91.511)	absence
	2. **pre-S1 del (7.310)**	2. pre-S1 del (nt 2854-3021) (5.744)	
	3. pre-S2 del (0.807)	3. pre-S2 del (nt 48-56) (0.294)	
	4. pre-S1+pre-S2 del (0.373)	4. pre-S1+pre-S2 del (nt 2855-2872, 1-54) (0.322)	

Table S1. List of the NGS-based pre-S genotyping result in 75 HBV-related HCC patients (continued)

Patient No.	Pre-S Del Type (%)[a]	Pre-S Del Region (%)[c]	Del Spanning Pre-S2 Gene Segment[d]
73	1. **wild-type (52.297)**	1. wild-type (52.297)	presence
	2. **pre-S1+pre-S2 del (31.339)**	2. pre-S1+pre-S2 del (nt 2855-2872, 3012-3086, 1-51) (8.935)	
	3. **pre-S1 del (12.863)**	3. pre-S1 del (nt 2855-2872, 3012-3092) (6.480)	
	4. pre-S2 del (3.502)	4. pre-S2 del (nt 1-51) (2.094)	
74	1. **wild-type (58.984)**	1. wild-type (58.984)	presence
	2. **pre-S2 del (34.533)**	2. pre-S2 del (nt 1-57) (20.926)	
	3. pre-S1+pre-S2 del (4.497)	3. pre-S1+pre-S2 del (nt 3026-3205, 2-55) (0.590)	
	4. pre-S1 del (1.986)	4. pre-S1 del (nt 2944-3120) (1.128)	
75	1. **wild-type (97.112)**	1. wild-type (97.112)	absence
	2. pre-S1 del (2.178)	2. pre-S1 del (nt 2854-3021) (0.641)	
	3. pre-S2 del (0.679)	3. pre-S2 del (nt 25-54) (0.355)	
	4. pre-S1+pre-S2 del (0.031)	4. pre-S1+pre-S2 del (nt 2855-2970, 25-54) (0.007)	

[a]The total percentage of pre-S gene DNA in each type of pre-S deletion was shown in descending order.

[b]The pre-S deletion type whose percentage was above the cut-off percentage (4.643) was shown in bold.

[c]The pre-S gene DNA with the highest frequency in each type of pre-S deletion was shown.

[d]The presence of deletion spanning pre-S2 gene segment was defined as the percentage of either pre-S2 or pre-S1+pre-S2 deletion above the cut-off percentage (4.643).

Abbreviations: NGS, next-generation sequencing; HBV, hepatitis B virus; HCC, hepatocellular carcinoma; del, deletion; nt, nucleotide.
